# A Call for Recruits from Clergy and People

**Published:** 1887-11-05

**Authors:** 


### The Hospital

A Weekly Institutional Journal of

Science, Medicine, and Philanthropy.

*For Hospitals, Asylums, Medical Practitioners, Students, Nurses, Families, Parishes, Congregations, and the Charitable Public.*

Vol. III. No. 58.] [November 5, 1887.

### A Call for Recruits from Clergy and People.

Food, religion, and medicine minister to three primary and fundamental necessities of man's nature. The demand for the first is the most prompt and imperious; but it is not more real or more universal than the instinctive desire and experienced need for the other two. Food must be obtained constantly, because hunger is constant and urgent. Religion and medicine are felt to be necessities quite as much, but the need for them asserts itself more decidedly in certain crises of man's experience rather than from day to day. All men have a general relation to these great facts, and every man has his own personal share of interest and responsibility in regard to each of them. The production of food and its preparation for use in civilised society are the work of particular classes, but that is only because of the general adoption of the principle of the division of labour, and not because other classes have no interest in the work. On the contrary, they have in it an interest of the most primary and fundamental character. The very same propositions hold good of religion and of medicine; and in the case of the latter we propose to show the general and unavoidable relation sustained by all men towards it, and the necessity that arises in a highly developed civilisation for each individual to recognise the relation, and voluntarily to give assent and effect to the claims implied by such a recognition.

What do we mean by the term "medicine" as here used? No person familiar with the literature of recent date can possibly regard medicine as identical with "the healing art." A century or two ago everywhere, and perhaps beyond the confines of civilisation even now, "medicine" and the "art of healing" were identical and convertible. But among civilised peoples something totally different is understood, at least by the educated, when the term is employed. Medicine is now in the fullest sense a "science of man." It can neither be comprehended scientifically nor practised perfectly on any narrower basis. Its field of study is man in his whole nature, development, history, and environment. Everything that man thinks; everything he does; everything that is done to him; every influence that bears upon him, nearly or remotely—these are the proper and essential conditions of his being, and these are therefore the proper and necessary studies of that separated class which aspires to act as a guiding providence to him from his first entry into the world to his departure from it. Medicine aims at gaining for man the fullest development of all his powers—physical, intellectual, moral. It proposes to itself to secure for him the most perfect armamentarium for assault and defence in the battle of life. It desires to be, in the picturesque language of holy scripture, a "shield and a buckler," a "tower of defence against all his enemies." It is all this, and the "healing art" as well.

If that be so—and who will deny it?—can it be thought by any intelligent person that he has given effect to every claim that medicine has upon him when he has regarded it as he regards a bowl of gruel or a mustard poultice—as something to be had recourse to if ever a painful necessity should arise, but as being no concern of his except in the event of such a necessity? As well might he say that the food supply, the education of his time, the provision of pure water and fresh air are no concern of his. We repeat with emphasis, and we think we have shown by argument, that every intelligent human being has a personal relation to medical science of a character which is not subsidiary and accidental, but primary and fundamental.

It is desirable and eminently necessary to bring every adult man into personal relation with the medical science and practice of his time, and that for two reasons among many others. Every man has something to gain from it, and it is important for him to be able to discern whether or not he is gaining the best it has to offer. That is one reason. But every man can contribute his share towards its development and progress by his personal conduct and his critical judgment, and that is a part of human duty which if left undone inflicts an injury upon medicine, as upon every other science. For medicine, like other special studies and arts, does not flourish best when left entirely in the hands of its priests and professors. There is such a thing as pedantry even among men of science; and unproved hypothesis has sometimes held long sway even over the minds of some of those who have most loudly proclaimed their unyielding loyalty to facts. The pure and free air of an universal criticism is as much needed for the perfect sanitation of medicine as it is for the purifying of religion or the growth and development of education.

So far as we are aware no effort has ever been made to bring the whole community into personal and immediate relation with what may be called the *cult* of physic. Such an effort is now to be put forth; and, if it be duly considered, it cannot but be productive of widespreading results of a highly practical character. The general hospitals may be regarded as the modern temples of the religion of medicine. And well do they deserve the name of temples, and the fame of a noble faith. Recent assaults made upon them, in books and elsewhere, have no solid foundation in fact and no justification in experience.

they will die like flames fed by a handful of thorns. The hospitals have great and impressive lessons to teach the men of this generation, and a bridge is wanted whereby all men may easily cross from their daily avocations to gain the instruction they have to give. They have also also great claims to make upon all men; and an ambassador is required who shall clearly make all men see not only the justice of the claims, but also the honour which is to be derived by the ready comprehension, and as ready response which every right-minded man may be expected to give.

The Hospitals Association has undertaken the task of striving to bring all men of all ranks and conditions into personal relation with the hospital system. It is appealing to the recognised leaders of the people, the aristocracy, the clergy and ministers of religion, the medical and other professions, the boards of guardians and all who, by virtue of inherited position or acquired influence, can in any way be considered guides to their fellows. A scheme has been carefully prepared whereby parishes, congregations, and individuals may become intimately associated with the hospital system, and may gradually obtain an easy and inexpensive, but full and complete education in all matters pertaining to hospital work and management.

That such a general association of the whole population with the hospitals would be fraught with very great advantages both to them and to the people, no one who has given time to the consideration of the subject for a moment doubts. A detailed account of the scheme by Sir Andrew Clark, Bart., M.D., is published on page 91, and has been sent to persons belonging to the classes named, and it is eminently to be desired that every one who receives the papers shall give to them that fair and intelligent consideration which the nature of the case demands.

The effort is in no sense an appeal for funds on behalf of any or of all hospitals. It is the beginning of a systematic movement to place hospitals on the broad base of the whole people's intelligent and benevolent regard. That it will, if successful, result in the prosperity and efficiency of the hospitals is undoubted, and such is the intention of those who have initiated the movement. But it is equally certain that its success would communicate a great impetus to medical science and practice in every part of the country, and by the gradual enlightenment of the public mind make ignorant and unskilled practice more and more difficult and more and more rare. By the universal success of the movement, the area of hospital resources would be so widened that the merest fraction of help from individuals would suffice to keep the whole system in full and growing efficiency, whilst the reflex advantages resulting from an increasingly intelligent knowledge of hospitals and their work would be of the highest service to the community. The hospitals would gain exactly that extended help without which they are now quite inadequate to the complete fulfilment of all their greatest duties: the people at large would gain a thousandfold more in the acquisition of that knowledge which in a highly civilised state is an essential condition of healthy and prosperous life. To the clergy and ministers of religion, who have unique opportunities of instructing the public in those broad general principles of social amelioration which are so well illustrated in hospitals, Sir Andrew Clark's scheme may be commended as a departure worthy of the most thoughtful consideration.

